# The Relationship Between Motor Competence Physical Activity Cardiorespiratory Fitness and BMI in UK Adolescents

**DOI:** 10.1080/02701367.2023.2265442

**Published:** 2023-10-24

**Authors:** Benjamin David Weedon, Patrick Esser, Johnny Collett, Hooshang Izadi, Shawn Joshi, Andy Meaney, Anne Delextrat, Steve Kemp, Helen Dawes

**Affiliations:** a Oxford Brookes University; b Drexel University; c Oxfordshire Sports Partnership; d University of Exeter; e NIHR Exeter Biomedical Research Centre

**Keywords:** Adolescents, balance, cardiorespiratory fitness, motor competence, physical activity

## Abstract

**Purpose:** This study set out to identify the extent of the relationships between subsections of the Movement Assessment Battery for Children 2nd Edition - MABC2 (manual dexterity, aiming and catching, and balance) to PA, CRF and BMI in adolescents. **Methods:** Height, BMI, the MABC2, a 20m shuttle run test and wrist-worn accelerometery PA levels (mins) were measured. Multivariable linear regression models, adjusting for sex, height and BMI were used to assess the relationship of the three subsections of the MABC2 with PA, CRF and BMI. **Results:** A total of 155 adolescents, aged 13-14 years, took part in this study (77 girls, 78 boys). Balance reported significant relationships with moderate to vigorous PA (unstandardised Beta B=0.15, 95%CI 0.02-0.28), vigorous PA (B=0.06, 95%CI 0.02-0.09) and BMI (B=-0.01, 95%CI -0.02-0.005). Balance in addition to aiming and catching skills were both significantly related to adolescent CRF (B=0.30, 95%CI 0.17-0.42 and B=0.29, 95%CI 0.14-0.45, respectively). **Conclusion:** This study suggests that balance is the strongest correlate skill to achieving the highest intensities of PA and healthier BMI status in adolescents.

In the UK, 78% of boys and 85% of girls fail to meet the physical activity (PA) guidelines of 60 min of moderate to vigorous physical activity (MVPA) per day (Bull et al., 2020; Wilkie et al., 2016). Evidence has shown a significant decrease in young people’s cardiorespiratory fitness (CRF) with a corresponding increase in obesity over recent years (Ng et al., 2014; G. Sandercock et al., 2010; G. R. Sandercock et al., 2015; Weedon et al., 2022). Increased periods of inactivity, obesity, and reduced CFR have shown a strong negative relationship with health outcomes persisting into later life (Garcia-Hermoso et al., 2020; Senechal et al., 2021). Developing effective strategies to promote children and adolescents’ PA and CRF is therefore urgently required.

One area, which has provided promising evidence for improving PA and CRF in young people, is Stodden’s conceptual model for increasing motor competence (MC) (Lima et al., 2017, 2018; Lopes et al., 2018; Robinson et al., 2015). MC is defined as goal-directed human movement (Robinson et al., 2015) and is suggested to have a reciprocal relationship with PA, which is mediated by CRF (Robinson et al., 2015; Stodden et al., 2008). Further models from De Meester and Stodden suggest that children who do not acquire adequate MC (De Meester et al., 2018) in movements such as throwing, catching, running, jumping, and dynamic balance are less likely to engage and maintain the recommended levels of PA (De Meester et al., 2018; Hulteen et al., 2018). Unfortunately, reduced levels of PA reduced the opportunity to increase or maintain their CRF and the health benefits it provides, such as optimal body composition.

The current literature indicates inconsistent relationships between MC, PA, and CRF in adolescents (Gisladottir et al., 2019; Lima et al., 2017; Logan et al., 2015; Ré et al., 2020). This may be partly explained by the movement tests used to assess MC. Currently, the Movement Assessment Battery for Children 2^nd^ Edition (MABC2) which, is a product-oriented, norm-referenced test, has been recommended by the European Academy of Childhood Disability (EACD) to assess MC disorders such as (DCD). However, like many other movement tests, (e.g. The Bruininks–Oseretsky Test of Motor Proficiency 2^nd^ edition—BOT2) (Bruininks, 2005) fine dexterity is measured and combined into a total overall score of MC along with balance and aiming and catching MC. This may weaken the relationship between MC, PA, and CRF as fine dexterity is not utilized in PA activities in adolescents (Holfelder & Schott, 2014; Loprinzi et al., 2015).

Currently, there is limited research exploring the extent to which different components of MC relate to different intensities of PA, CRF, and obesity in adolescents. We propose to use the subsections of the MABC2 to explore this relationship. We hypothesize that balance and aiming and catching will show a greater relationship to PA and CRF compared to manual dexterity as this is not a prominent motor competency required for sports or exercise-based activities (Holfelder & Schott, 2014; Loprinzi et al., 2015). Therefore, we set out to explore the extent of the relationship between each component of MC with different intensities of PA, CRF, and BMI status in adolescents.

## Method

### Study design

Data were collected as part of a convenient sample for the cross-sectional screening process for the Rhythmic Motor Learning in Children with Developmental Coordination Disorder study (Clincaltrials.gov NCT03150784). All parents or legal guardians consented for their children to take part in this study. Oxford Brookes University Ethics Committee (UREC 161033) granted ethical approval.

### Participants and setting

Participants aged 13–14 years were recruited from one comprehensive secondary school between September 2017 and July 2018 in the UK. The catchment area for this school ranges from the first to the third quintile on the Index of Multiple Deprivation (IMD) score (National Perinatal Epidemiology Unit NPEU, Nuffield Department of Population Health, 2013). No participants were formally diagnosed with Developmental Coordination Disorder (DCD), but 40 participants scored ≤5 for the MABC2 total score. Inclusion criteria consisted of adolescents attending a comprehensive secondary school and aged 12–15 years. Participants were excluded if they had any known medical condition that could explain deficits in walking, MC, or movement, as described by the DSM-5 (DSM-5, 2013). Parents provided this information through a physical health questionnaire (Physical Activity Readiness Questionnaire) and exclusion criteria check sheet, which was also checked against the school’s medical records.

### Variables and measurements

Measurements of MC and CRF were collected during a timetabled PE lesson. This was conducted in a circuit-style format, where groups consisting of 3–4 students performed one station at a time and rotated around each station (12 stations in total). Half the sample performed the MC tasks first, while the other half performed the CFR test. This was to mitigate any potential order effect. Data were collected by physiotherapist, nurses, research staff, and health-care PhD students. All were trained to assess the measures described. Further details have been discussed in the previous work (Weedon, 2020).

### Quantitative variables

#### Anthropometrics

Height and weight were measured with a portable Harpenden Stadiometer (Holtain, Crymych, UK) and a SECA medical 770 digital floor scale (SECA, Hamburg, Germany), respectively. Participants were dressed in light sports clothing and were instructed to remove their shoes. Height and weight were used to calculate the body mass index (BMI), which is presented as age- and sex-independent z-scores (WHO) (de Onis et al., 2007).

#### Motor competence (MC)

The Movement Assessment Battery for Children, 2nd edition (MABC2) was used to assess overall MC, which consists of three sub-sections manual dexterity, aiming and catching, and balance (two board balance, walking backwards heel to toe, and zig-zag hopping), for 11–16 years (Henderson et al., 2007). Individual raw scores were converted into individual sub-section scores (Henderson et al., 2007).

#### Cardiorespiratory fitness

CRF was assessed using the 20 m shuttle run test (20mSRT) (Léger & Lambert, 1982; Leger et al., 1988). Participants were instructed to run back and forth between two markers that were 20 m apart. The time required to run between each marker became shorter as the test progressed, requiring participants to run faster. The participants were withdrawn from the test when they failed to reach the marker within the allotted time on three consecutive occasions, or they withdrew from the test themselves. The total number of shuttles that they achieved was recorded as their score (Ramsbottom et al., 1988).

#### Physical activity (PA)

PA was measured using a wrist-worn accelerometer (Axivity AX3) over 7 days which has shown good accuracy in determining physical activity intensities (Doherty et al., 2017; Hedayatrad et al., 2020). Acceleration (±8 g) was measured in three planes: vertical, medial-lateral, and anterior-posterior at 100 Hz. These accelerations were then transformed into a single vector magnitude [g] and reduced into one-second epochs [gs], using a customized programme in LabVIEW 2015 (National Instruments Austin, USA). The data were analyzed over 14 h, from 08:00 to 22:00 (Fairclough et al., 2016). A set of minimum wear-time criteria were applied to standardize a valid day. These included a minimum of 8 h of wear time per day for at least three weekdays and one weekend day (Weedon, 2020). The non-wear time was calculated as consecutive time spent in the sedentary category (<6 gs) for ≥60 min. From this, cut points were applied to categorize time [min/day] spent in moderate (MPA 22–56 gs), and vigorous PA (VPA >56 gs). Moderate to Vigorous PA (MVPA) was calculated as the sum of MPA and VPA according to methods published by Phillips et al. (2013).

#### Statistical analysis

Descriptive data are presented as means and standard deviations. All data were assessed for normality using histogram plots. Normally distributed data used linear regressions to assess the extent to which physical activity, cardiorespiratory fitness, and BMI z-scores were dependent on manual dexterity, aiming and catching, and balance subsection scores. All linear models incorporated the three sub-sections of the MABC2 and were adjusted for sex. MVPA (model 1) and VPA (model 2) were further adjusted for BMI z-score. Total shuttles were adjusted for BMI z-score and height (model 3). Little’s chi-squared test for data missing completely at random (MCAR) was used to assess the missingness of the data for the whole dataset (Little, 1988). The data were assessed and reported to have no multicollinearity or homoscedasticity. These data were analyzed by StataCorp. 2019. Stata Statistical Software: Release 16. College Station, TX: StataCorp LLC, with *p*-value set at < .05.

## Results

**Figure 1.**
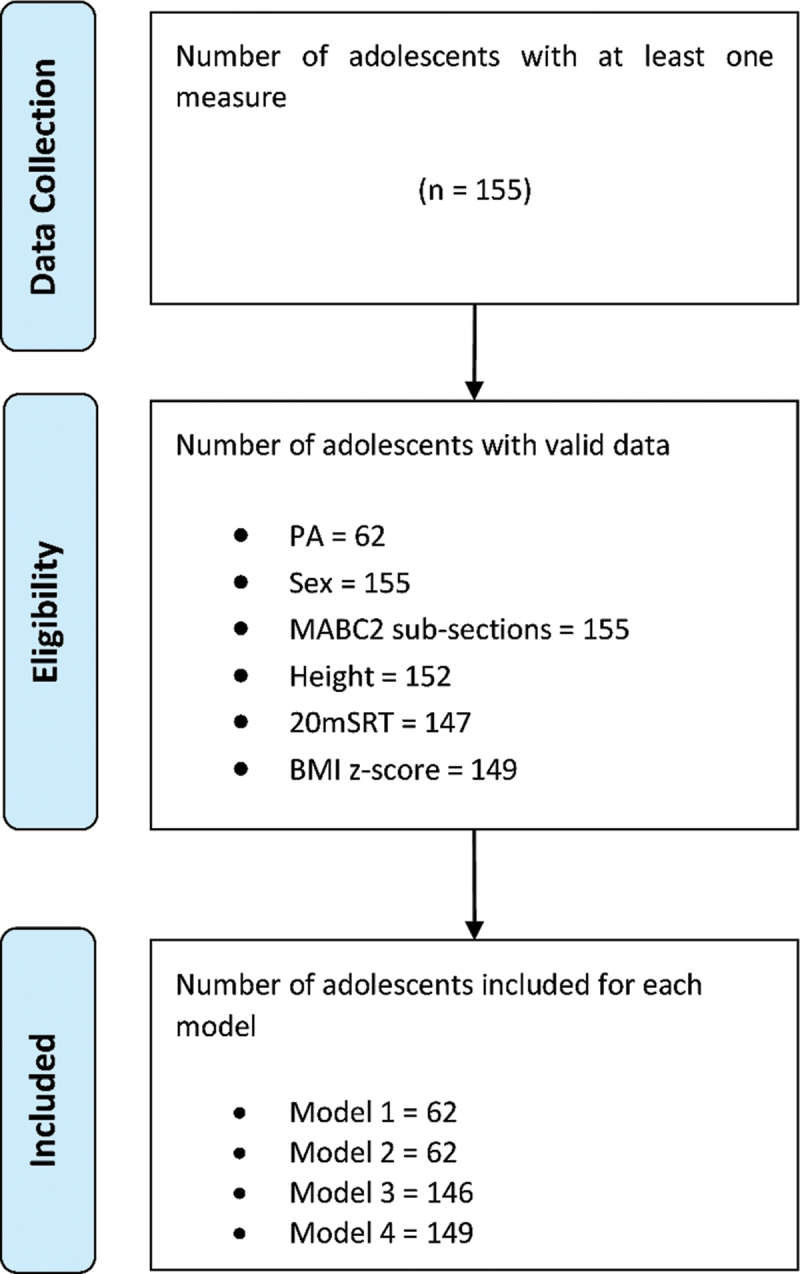
Flow diagram for the number of adolescents included in the study. (20mSRT = 20m shuttle run test, PA = physical activity, BMI = body mass index, MABC2 = Movement Assessment Battery for Children 2nd edition).

A total of 155 students were screened for this study, and their summary statistics are presented in Table 1. There were significant differences between sexes, with boys being taller and scoring higher on the total shuttles test, and aiming and catching. Girls reported a higher score for manual dexterity compared to the boys (Table 1). The adolescents in this sample were classed as healthy weight as described by their BMI z-score (de Onis M et al., 2007) and had above average fitness levels (70^th^−80^th^ percentile) (Tomkinson et al., 2017). Physical activity data from (93) participants were excluded as they did not meet the minimum required wear time. Missing data were also reported for the 20 m shuttle run test (8), height (3), and BMI z-score (6), which resulted in the analysis of 62 participants’ data for models one and two (Table 2). As models three and four did not incorporate PA analysis, the total samples were 146 and 149, respectively (Figure 1). We tested for data MCAR and showed that the missingness of data was random (*p* = .54). This sample consisted of a wide range of MC abilities; however, only one participant managed to average more than 60 min of MVPA per day.

**Table 1.** Summary characteristics.

	Boys				Girls			
	n	mean (SD)	95% CI	min-max	n	mean (SD)	95% CI	min-max
Height [cm]*	78	168.9 (9.3)	166.8–171	136–194	74	162.2 (9.6)	160–164.5	107–181
Weight [kg]	78	58.1 (14.5)	54.8–61.4	29.8–106	73	57.2 (12.9)	54.2–60.2	30.3–90.6
BMI [z-score]	77	0.5 (1.3)	0.2–0.8	−2.3–3.4	72	0.7 (1.4)	0.3–1	−4.7–4.9
MVPA [mins/day]	32	29.7 (12.4)	25.3–34.2	5.6–56.6	30	28.3 (16.2)	22.2–34.3	5.2–62.2
VPA [mins/day]	32	5.4 (4.1)	3.9–6.9	0.3–18.2	30	3.9 (4.1)	2.3–5.4	0.2–17.3
Wear time [hrs/day]	32	10.8 (1.8)	10.2–11.5	6.4–13.9	30	10.9 (2.1)	10.1–11.7	7.0–13.9
Days worn [count]	32	6.2 (1.1)	5.8–6.6	4–7	30	6.6 (1.4)	6.0–7.0	4–7
Total shuttles [count]*	76	56 (25)	50.3–61.7	18–118	71	41 (22.8)	35.8–46.6	12–110
MABC2 [percentile]	78	23.9 (20.8)	19.2–28.6	0.5–84	77	18.7 (18)	14.6–22.8	0.1–75
Balance score [percentile]	78	36 (27.7)	29.8–42.2	0.5–91	77	33.1 (30.6)	26.2–40.1	0.5–91
Aiming and catching score [percentile]*	78	53.5 (25.8)	47.6–59.3	0.1–98	77	21.9 (20.5)	17.3–26.6	0.1–84
Manual dexterity score [percentile]*	78	16.2 (17.6)	12.2–20.1	0.1–84	77	24.7 (21.7)	19.8–29.6	0.5–84

*Note*. MVPA = moderate vigorous physical activity, VPA = vigorous physical activity, MABC2 = Movement Assessment Battery for Children, 2nd edition, SD = standard deviation, 95% CI = 95% confidence intervals, min-max = minimum to maximum values, * = *p* < .01.

**Table 2.** Multivariate regression model for three subsections of motor competence to MVPA, VPA, total shuttles, and BMI z-score.

MVPA^a^	B	β	95% CI B		*p*
n = 62			LL	UL	
**Model 1**					
Balance score	0.15	0.31	0.02	0.28	.02
Manual dexterity score	−0.03	−0.04	−0.22	0.16	.75
Aiming and catching score	−0.05	−0.10	−0.19	0.09	.45
VPA^a^	B	β	95% CI B		*p*
n = 62			LL	UL	
**Model 2**					
Balance score	0.06	0.41	0.02	0.09	.002
Manual dexterity score	−0.04	−0.20	−0.09	0.01	.12
Aiming and catching score	0.007	0.05	−0.03	0.05	.69
Total shuttles^b^	B	β	95% CI B		*p*
n = 146			LL	UL	
**Model 3**					
Balance score	0.30	0.34	0.17	0.42	5.2×10^−6^
Manual dexterity score	−0.16	−0.13	−0.34	0.02	.09
Aiming and catching score	0.29	0.33	0.14	0.45	.0002
BMI z-score^c^	B	β	95% CI B		*p*
n = 149			LL	UL	
**Model 4**					
Balance score	−0.01	−0.26	−0.02	0.005	.001
Manual dexterity score	−0.008	−0.12	−0.02	0.002	.14
Aiming and catching score	0.006	0.13	−0.003	0.02	.19

*Note*. B = beta coefficient, CI = confidence intervals, LL = lower limits, UL = upper limits, β = standardized beta, *p* = significance value, MVPA = moderate to vigorous physical activity. ^a^ = adjusted for sex and BMI z-score, ^b^ = adjusted for sex, BMI z-score, and height, ^c^ = adjusted for sex.

Table 2 reports the multivariate linear models for the three sub-sections of the MABC2 predicting MVPA, VPA, total shuttles, and BMI z-score. Balance was the only significant predictor for MVPA, VPA, and BMI z-score (models 1, 2 and 4). For model three balance and aiming and catching scores both significantly predicted total shuttles. However, balance reported a slightly higher standardized beta coefficient compared to the aiming and catching score. For models where the sub-sections were predicting MVPA, VPA, and total shuttles, the linear relationships were positive indicating that increased balance predicted increased levels of PA and CRF. Model four indicates an inversely proportional relationship between balance and BMI z-score where increases in balance predicts a lower BMI z-score.

## Discussion

This study presents data on the relationship between individual measures of motor competence to physical activity, cardiorespiratory fitness, and BMI status that is currently lacking in adolescents. We found evidence that balance as measured by the MABC2 is related to MVPA, VPA, CRF, and BMI, whereas other components such as manual dexterity did not, which may provide a masking effect. Our findings support research into the training of balance as an intervention to determine the impact on PA, fitness, and BMI in adolescents. It may also provide a quick objective measure for physical educators to assess students who may be at risk of poor health and fitness alongside its potential for providing a targeted intervention to reverse these negative health trends.

Previous models explaining the relationship between motor competence (MC) and physical activity (PA) are described as reciprocal, whereby a positive feedback loop exists between these two variables, which is further mediated by cardiorespiratory fitness (CRF) and inversely related to BMI status (Lima et al., 2017; Stodden et al., 2008). Our current study has been able to provide evidence that balance skill was only significant predictor for moderate to vigorous PA (MVPA) and, more importantly, higher levels of PA intensity (VPA) in adolescents (DuBose et al., 2018). Previous longitudinal studies have reported similar positive associations with baseline MC and follow-up PA duration (Lima et al., 2017; Lopes et al., 2018). Several adjusted models reported significant relationships between MC measured at baseline and time spent in moderate PA, MVPA, and total PA at follow-up. Interestingly, the previous study used the Körperkoordination-Test-für-Kinder (KTK) to assess MC, which only assesses balance MC (Lopes et al., 2018). Our study was able to highlight that balance is the most strongly related MC skill required for higher levels of PA and intensity when compared to other aspects of MC. However, these relationships can only be interpreted when the MABC2 was used as a measure of MC. Different measures of MC that utilize different movement tests may report different relationships. Our current study also uniquely predicts that higher levels of balance MC from MABC2 indicate higher durations spent in VPA, which is non-significant in the previous studies. The differences in these results may be explained by the differences in data processing of the PA data. The previous study epoched the data into two-second averages (Lopes et al., 2018), whereas, our current study epoched the data into one-second averages. These differences in epochs have been shown to result in different VPA durations with 1-s epochs reporting more accuracy when assessing VPA in children and adolescents (Aibar et al., 2015; Fabre et al., 2020; Fröberg et al., 2017). Currently there is no consensus for epoch duration or intensity threshold when analyzing children and adolescents’ PA (Banda et al., 2016). Further research should aim to provide consistent methods for PA analysis as this would allow for better comparisons between studies.

Cardiorespiratory fitness (CRF) is declining significantly in adolescents over previous years (Weedon et al., 2022) and increasing efforts have been made to try and reverse this worrying health trend. Our current data show that aiming and catching skills and balance were positively associated with higher CRF in adolescents. This supports recent evidence that has assessed the longitudinal relationship between motor competence and fitness (Lima et al., 2018). Previous data have reported that children with low MC, as measured by the KTK, indicate a higher risk of low CRF in adolescence (Lima et al., 2018). Interestingly, this study reported that MC had a greater effect on future CRF compared to the reversed causality of CRF affecting future MC levels. Our current study also provides evidence that aiming and catching skills may also be an important factor to promote CRF in adolescents. This evidence supports previous findings that higher levels of object control (kick, catch, and throw) in children are associated with higher CRF in adolescents (Barnett et al., 2008). This may be explained by the association between these types of MC skills and vigorous forms of PA. Recent evidence has indicated that higher intensities of PA produce higher levels of CRF in adolescents (Burden et al., 2022). These higher levels of PA intensities are most commonly performed in sports and game-based activities, which specifically require high levels of balance and aiming and catching MC (Barnett et al., 2008). Therefore, adolescents who possess high MC that is required for higher intensities of PA are more likely to report higher levels of CRF (Robinson et al., 2015; Weedon et al., 2018), which is supported by our current study. It is then important for MC skills such as balance, aiming and catching, and object control to be assessed, monitored, and targeted for improvement throughout childhood and into adolescents, to maximize the potential benefits associated with PA and CRF (Weedon et al., 2022).

The relationship between MC and BMI status is complicated, as BMI has been described as a consequence of low MC and a contributor to children’s and adolescents’ low MC levels (Stodden et al., 2008). However, current research has suggested that interventions targeting improvements in BMI status should involve increasing MC (Truong et al., 2021). Our study shows that balance from the MABC2 subsection is the only significant predictor of BMI status when compared to other subsections of MC and may provide a more targeted approach when MC is considered as part of an intervention. Previous studies have reported significant relationships between MC and BMI status in children and adolescents (Chagas et al., 2021; Lima et al., 2018), which has indicated adolescents with low levels of MC are at six times greater risk of being overweight or obese than adolescents with typical levels of MC (Chagas et al., 2021). However, none have assessed which subsection of MC is driving this relationship with many studies only focussing on one aspect of MC (Chagas et al., 2021; D’Hondt et al., 2014; Lima et al., 2018).

Even though our data indicate that balance MC is related to BMI status, it could be that this relationship is a result of PA. We know that PA duration and intensity are largely responsible for controlling healthy BMI status (Videira-Silva et al., 2020) and greater PA durations and intensities are strongly linked to balance MC, then we may be indirectly measuring the relationship of PA on BMI status. However, previous longitudinal data have shown that MC presented a direct relationship and was mediated by VO_2peak_ with body fatness, whereas, PA only influenced body fatness through mediation with VO_2peak_ (Lima et al., 2017). They concluded that MC and VO_2peak_ expressed a greater effect on body fatness compared to PA in adolescents over time. This provides evidence that balance MC directly affects BMI status in adolescents and should be used in interventions to combat the increasing obesity prevalence.

There are some notable limitations related to this study. The cross-sectional study design limits the ability to infer causality. However, previous longitudinal studies have reported the effect of MC on physical activity (Lopes et al., 2018), cardiorespiratory fitness (Lima et al., 2018) and BMI status (D’Hondt et al., 2014; Lima et al., 2018) in adolescents and have shown that it has a positive effect on health. The large amount of missing PA data may cause a bias and increase type one errors. Even though there was a relatively large amount of PA data that did not meet the valid wear time requirements, we were able to prove that the missingness of data was missing completely at random. There is also a large range of movement assessments designed for different ages that utilize different movement tests to assess MC. As there is no current gold standard for MC assessment in adolescents, it is difficult to compare these results to other studies and may indicate the inconsistencies throughout the literature. BMI has limitations in its ability to assess body composition compared to more direct measures. However, the BMI z-scores from the WHO (de Onis et al., 2007) provide a large sample for specific ages. Finally, the relatively small sample size limits the generalizability of these results in the wider population.

We support the current evidence that MC is a vital part of healthy physical development from childhood into adolescence and a focus on MC, especially balance MC, should be targeted for monitoring and interventions when PA, CRF, and BMI do not meet the recommended guidelines.
